# Detection of Rotavirus Using Padlock Probes and Rolling Circle Amplification

**DOI:** 10.1371/journal.pone.0111874

**Published:** 2014-11-04

**Authors:** Anja Mezger, Christina Öhrmalm, David Herthnek, Jonas Blomberg, Mats Nilsson

**Affiliations:** 1 Science for Life Laboratory, Department of Biochemistry and Biophysics, Stockholm University, Solna, Sweden; 2 Section of Clinical Virology, Department of Medical Sciences, Uppsala University Hospital, Uppsala, Sweden; Catalan Institute for Water Research (ICRA), Spain

## Abstract

Rotavirus infections are one of the most common reasons for hospitalizations due to gastrointestinal diseases. Rotavirus is often diagnosed by latex agglutination assay, chromatography immunoassay, or by electron microscopy, which are all quite insensitive. Reverse transcription polymerase chain reaction, on the other hand, is very sensitive to variations at the genomic level. We developed a novel assay based on a set of 58 different padlock probes with a detection limit of 1,000 copies. Twenty-two patient samples were analyzed and the assay showed high concordance with a PCR-based assay. In summary, we present a new assay for sensitive and variation tolerant detection of rotavirus.

## Introduction

Rotavirus infections are one of the most common infections seen in young children. By the age of five nearly 100% of all children, regardless of ethnicity or socio-economic status, have been infected at least once with rotavirus [1]. Prior to vaccine introduction, rotavirus accounted for 36% of hospitalizations due to gastro-intestinal diseases [2] and about 611,000 children below the age of five died every year due to a rotavirus infection [3]. Vaccine introduction has substantially decreased the magnitude of rotavirus infection in the United States [4]. A simple and fast diagnostic test is needed in order to control disease outbreak and spread as well as for epidemiological disease surveillance purposes.

Currently, enzyme immunoassays, latex agglutination assays, electron microscopy and reverse transcription polymerase chain reaction (RT-PCR) are commonly used for detection of rotavirus. However, all these tests have drawbacks. Latex agglutination assays and electron microscopy lack sensitivity [5], [6] and in addition, electron microscopy is very labor-intensive and requires highly trained personnel which makes it unsuitable for routine diagnostics. RT-PCR, on the other hand, has a high sensitivity, is relatively inexpensive, but islimited in its multiplexity and its variation tolerance. Newly occurring variants can be missed by PCR due to mismatching primers.

Here we present a new sensitive and rapid method for the detection of rotavirus, a dsRNA virus, from fecal samples. It is based on a modified version of a padlock probe-based assay targeting the ssRNA Crimean–Congo hemorrhagic fever virus [7]. In the present rotavirus assay, we target an even higher sequence complexity of detected strain variants, and we use a digital readout. The approach is nucleic acid variation tolerant and detects a broad range of different rotavirus strains. To achieve this, the assay uses padlock probes that can be highly multiplexed. Padlock probes are linear oligonucleotide probes which have target-complementary ends and circularize upon correct target recognition [8]. The two target-complementary ends are connected via a backbone that includes a restriction site and a tag sequence for detection. Once the ends of a padlock probe are ligated, the circle is amplified by rolling circle amplification (RCA) [9]. The rolling circle products (RCPs) are monomerized and religated to allow for a second round of RCA which additionally increases amplification 300 times [10]. RCPs are visualized by hybridizing short complementary oligonucleotides coupled to a fluorophore to the tag sequence [11]. This allows detection in a microfluidic setup in a confocal microscope [11].

In this study, we successfully detected rotavirus, a highly variable dsRNA virus, with good sensitivity.

## Materials and Methods

### Patient Samples

Rotavirus positive samples were previously detected with rotavirus antigen detection by enzyme-linked immunosorbent assay (ELISA) or 7-plex gastro VOCMA (multiplex PCR and liquid bead array), and the target region was further sequenced with a 3130 Genetic Analyzer (Applied Biosystem) [12]. Sequence information was obtained for all samples except sample 1, 14 and 20. Out of the 17 sequenced samples 8 different genotypes were present (Figure S1).

The fecal samples were collected and stored with the patients consent according to the Swedish Biobank Law (SFS 2002∶297) and were analyzed anonymously. All samples have been described in Öhrmalm *et al.* [12].

### Probe design

#### Design of padlock probes

**Figure 1:**
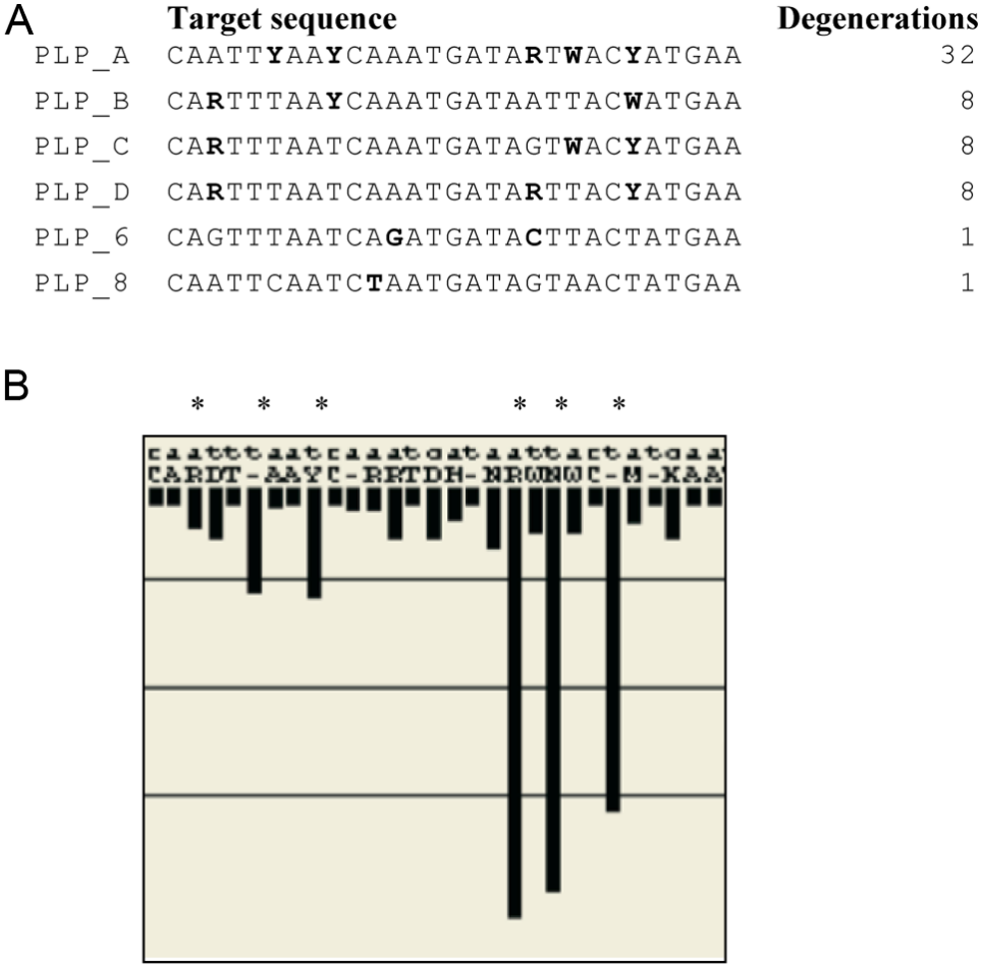
Padlock probe design. (A) The sequence of the six padlock probes with the haplotyped degenerations and ligation site. (B) Alignment of 214 Human Rotaviruses. The variations in Rotavirus were mapped using BLASTn and ConSort. The length of the black bars represents the frequency of variation as an average percentage conservation at each nt position (y-axis).

All Human Rotavirus A types that were available on NCBI blastn (National Library of Medicine) were used to design the padlock probes. Using the Consort program [12], sequence conservation and frequencies of nucleotide variation were compared for all eleven human Rotavirus A segments for all available sequences in NCBI blastn (National Library of Medicine). This resulted in that the VP6 gene, coding for the major capsid protein, was chosen as a target region for the padlock probes based on its relative conserved regions. The chosen region, nt 96–125 of the 1356 nt long VP6 gene (Genebank nr AB022768), in which the padlock probes are designed against is based on the alignment of 214 human rotavirus A sequences (Figure 1B). A query of VP6 gene rotavirus in BLASTn was analyzed in the ConSort program [12] to construct a primer and the 5′ and 3′ arms of padlock probes targeting rotavirus. The design was made according to the principle of NucZip [13]. The consensus sequence for the target region in the NIH database is CARTTYAAYCWRATGATARTWACHATGAA, causing a degeneration of 384. Due to that specific nucleotide positions vary together, Consort settings of 1–10 degeneration were used to haplotype the padlock target region into a minimum of sequences. The final design consisted of six padlock probe designs (degeneration 32, 8, 8, 8, 1, and 1) that together create a mix of 58 unique padlock probes (Figure 1A) needed to cover 95% of the published sequences in the NIH database targeting both genogroups, I and II, of VP6. Further, the padlock probes (69 nt) sequence consist of a 5′ arm of 15 nt Rotavirus targeting probe, followed by the AluI restriction site, the target region for the detection probe, and 3′ arm of 14 nt Rotavirus targeting probe, and were ordered from IDT. All oligonucleotide sequences used in this assay are listed in Table 1.

**Table 1:** List of oligonucleotides used in this assay.

Name	Sequence^a^
cDNA primer	5′-Biotin-TGYARRTTCC AR+TTY+TCD+AT RTA-3′
Forward primer	5′-GGCTTTW+AAA+ CGAA+GTC+TTC R-3′
PLP_A	5′-**GATARTWACY** **ATGAA**GTGTA TGC*AGCT*CCT CAGTAGTGCG ACACATGACA TCAAC **CAATT YAAYCAAAT**-3′
PLP_B	5′-GATAATTACW ATGAAGTGTA TGCAGCTCCT CAGTAGTGCG ACACATGACA TCAACCARTT TAAYCAAAT-3′
PLP_C	5′-GATAGTWACY ATGAAGTGTA TGCAGCTCCT CAGTAGTGCG ACACATGACA TCAACCARTT TAATCAAAT-3′
PLP_D	5′-GATARTTACY ATGAAGTGTA TGCAGCTCCT CAGTAGTGCG ACACATGACA TCAACCARTT TAATCAAAT-3′
PLP_6	5′-GATACTTACT ATGAAGTGTA TGCAGCTCCT CAGTAGTGCG ACACATGACA TCAACCAGTT TAATCAGAT-3′
PLP_8	5′-GATAGTAACT ATGAAGTGTA TGCAGCTCCT CAGTAGTGCG ACACATGACA TCAACCAATT CAATCTAAT-3′
Restriction oligo	5′-GTGTATGC*AG CT*CCTCAGTA-3′
Detection oligo	5′- Cy3-GTTGATGTCA TGTGTCGCAC-3′
Target_6	5′-Biotin-TTCATAGTAA GTATCATCTG ATTAAACTG-3′
Target_1	5′-Biotin-TTCATAGTAA TTATCATTTG GTTAAATTG-3′

a A + sign before nt indicate the LNA residues. As implicated in PLP_A: the bold font indicated the Rotavirus targeting padlock probe arms, Italic font indicates the AluI restriction site, and the underlined sequence the sequence for detection probe hybridization.

#### RNA extraction

A suspension of approximately 100 µl feces in 1 ml of TE- buffer was vortexed and centrifuged. 400 µl of supernatant was transferred into 2 ml of EasyMag lysisbuffer and total nucleic acid was extracted with the NucliSENS EasyMag extraction system (bio-Mérieux AB, Sweden) into an eluate of 110 µl, which was aliquoted and frozen at −70°C.

#### Synthesis of biotinylated cDNA

Two µl of nucleic acid extract was mixed with 2.2 µM of the biotinylated reverse primer in a total volume of 9 µl. The biotinylated RT-PCR reverse primer (nt position 448–426) Biotin-TGYARRTTCCAR+TTY+TCD+ATRTA (+ before nt indicates locked nucleic acid (LNA) residues) was ordered from Exiqon (Exiqon A/S, Denmark). The LNA residues were included to increase binding stability of the primer. To denature the dsRNA the samples were incubated for 5 min at 97°C followed by snap-cooling on ice for 2 min. The reverse transcription was performed in a total volume of 20 µl in 1x First strand buffer (Life Technologies), 2 mM dNTPs (Thermo Scientific), 5 mM DTT (Life Technologies), 40 U RNaseOUT (Life Technologies) and 200 U Superscript III (Life Technologies) at 50°C for 60 min. The reaction was inactivated by heating at 70°C for 15 min.

The presence of cDNA was analyzed by PCR using 200 nM of forward primer (nt position 1–23) GGCTTTW+AAA+CGAA+GTC+TTCR (+ before nt indicate the LNA residues) together with 200 nM of the biotinylated reverse primer, resulting in amplicons of 448 nt. Additionally, the PCR reaction contained 2 µl of first strand reaction, 2.5 mM MgCl_2_, 0.8 nM dNTP (Applied Biosystem), 1x buffer and 0.8 U of AmpliTaqGold (Life Technologies), in a total volume of 25 µl. The reactions were run at 94°C for 10 min, 50 cycles at 94°C for 30 s, 52°C for 30 s and 72°C for 30 s, ending with 72°C for 7 min. Products were analyzed with 1.5% EtBr agarose gel.

#### Circle-to-circle amplification

Prior to use, padlock probes were phosphorylated at the 5′ end. Padlock probes were incubated in a mixture of 10 U T4 Polynucleotide Kinase (Thermo Scientific), 50 mM Tris-HCl (pH 7.6 at 25°C), 10 mM MgCl_2_, 5 mM DTT, 0.1 mM spermidine and 1 mM ATP for 30 min at 37°C followed by 20 min at 65°C. Padlock probes were ligated onto their target by incubating a mixture of 2.5 µl of sample cDNA, 50 nM of each padlock probe, 0.2 µg/µl BSA (NEB), 5 U Ampligase (Epicentre) and 1x Ampligase reaction buffer (20 mM Tris-HCl (pH 8.3), 25 mM KCl, 10 mM MgCl_2_, 0.5 mM NAD, and 0.01% Triton X-100) at 50°C for 10 min. Prior to use, Dynabeads MyOne Streptavidin T1 beads (Life Technologies) were washed 3 times with washing buffer (10 mM Tris-HCl (pH 7.5), 5 mM EDTA, 0.1% Tween 20, and 0.1 mM NaCl). Ten µl of 10 mg/ml Dynabeads were added to the ligated samples and incubated at room temperature for 5 min. All samples were washed once with washing buffer to remove unligated padlock probes. To replicate ligated circles, the washing buffer was discarded and replaced by a solution consisting of 0.2 µg/µl BSA, 125 µM dNTPs (Thermo Scientific), 100 mU/µl phi29 DNA polymerase (Thermo Scientific), and 1x phi29 DNA polymerase reaction buffer (33 mM Tris-acetate (pH 7.9), 10 mM Mg-acetate, 66 mM K-acetate, 0.1% Tween 20, 1 mM DTT). Polymerization occurred at 37°C for 20 min and was terminated at 65°C for 1 min. To monomerize the amplified RCPs, 5 µl of digestion mix (0.2 µg/µl BSA, 1x phi29 DNA polymerase reaction buffer, 0.6 U/µl AluI (NEB), and 600 nM restriction oligo) was added to the amplified RCPs and incubated at 37°C for 1 min followed by an inactivation step at 65°C for 1 min. Monomers were religated and a second round of RCA was carried out by adding 25 µl of 0.2 µg/µl BSA, 1.36 mM ATP, 28 mU/µl T4 DNA ligase (Thermo Scientific), 1x phi29 DNA polymerase reaction buffer, 100 µM dNTPs, and 120 mU/µl phi29 DNA polymerase. The mixture was incubated at 37°C for 20 min, followed by 65°C for 1 min. Undigested restriction oligos served as a ligation template and primer for the second RCA.

#### Visualization of rolling circle products

Cy3 coupled detection oligos were hybridized to the rolling circle products. Fifty µl of a labeling solution containing 40 mM EDTA, 40 mM Tris-HCl (pH 7.5), 0.2% Tween 20, 10 nM detection oligo, and 2 M NaCl was added and incubated at 70°C for 2 min, followed by 55°C for 15 min. Labeled rolling circle products were pumped through a micro-channel and visualized using a confocal microscope [11]. Samples used for evaluating the padlock probes were diluted 1∶10 in labeling solution before measuring.

## Results

**Figure 2:**
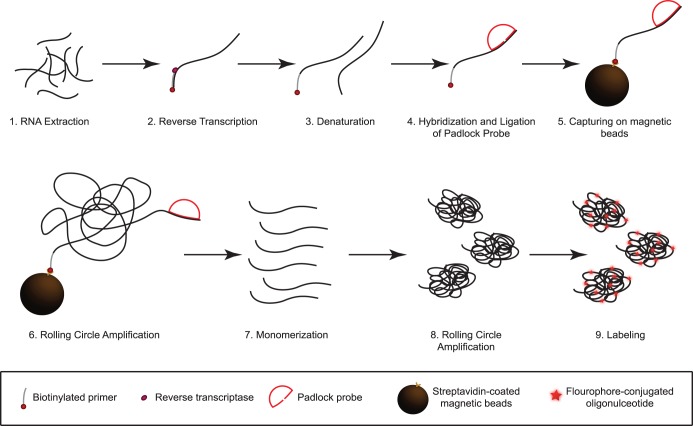
Schematic summary of assay principle. (1) RNA is extracted from patient samples and (2) cDNA is synthesized using biotinylated primers. (3) The DNA is denatured and (4) padlock probes are hybridized and ligated to their complementary target sequence. (5) This complex is captured onto magnetic beads to wash away unbound probes and (6) amplified by rolling circle amplification (RCA) for 20 min. (7) The rolling circle products (RCPs) are monomerized and (8) a second round of RCA is performed. (9) Fluorescently labeled oligonucleotides are hybridized to the RCPs for detection in a microfluidic setup using a confocal microscope.

The reaction scheme consists of several steps (Figure 2). Total nucleic acid is extracted from fecal samples, denatured (1), and cDNA is synthesized using 5′ biotinylated rotavirus specific primers (2). The biotinylated cDNA is denatured from the RNA strand (3). Padlock probes are hybridized and ligated onto their complementary target (4) and subsequently captured on magnetic beads to allow washing away excess padlock probes in the next step which otherwise would inhibit the following RCA reaction (5). Excess padlock probes are washed away. Upon successful ligation, padlock probes are amplified by RCA (6). To further increase the number of amplification products, RCPs are monomerized (7) and religated to be further amplified by RCA (8), a process referred to as circle-to-circle amplification (C2CA) [10]. This step allows for an extra 300-fold amplification of the target molecules. Finally, RCPs are labeled using a short complementary fluorescent oligonucleotide (9) and detected using a fluorescence-based microfluidic setup, permitting digital enumeration of RCA products [11]. In this setup the sample is pumped through a microfluidic PDMS chip placed on a confocal microscope. RCPs will appear as bright fluorescent object and can be enumerated using MATLAB.

### Evaluation of padlock probes

Since rotavirus is a highly variable virus it is important to cover as many strain variants as possible in order to achieve an accurate diagnosis with a low false-negative rate which might be caused by non-matching padlock probes. Our approach uses a mix of padlock probes which all target the same region in the VP-6 gene. These padlock probes differ only in a few bases and are therefore able to compete with each other by binding to the same region. In cases of imperfect binding, especially at the end of the probe arms, ligation would be hampered and no rolling circle product would be generated from these targets. To investigate if this competition and inhibition actually occurs, thus, if an increase in the number of unique padlock probes results in a decrease in signal, we serially added the different batches of padlock probes to one amol of synthetic target (5′-Biotin-TTCATAGTAA GTATCATCTG ATTAAACTG-3′) and performed C2CA. Importantly, we did not observe a lower amount of RCPs with an increasing number of padlock probes (Figure S2). Hence, there is no evident inhibition in the hybridization and ligation step by competing, non-matching padlock probes. These results indicate that probably even a larger degree of multiplexing is possible.

### Analytical sensitivity

**Figure 3:**
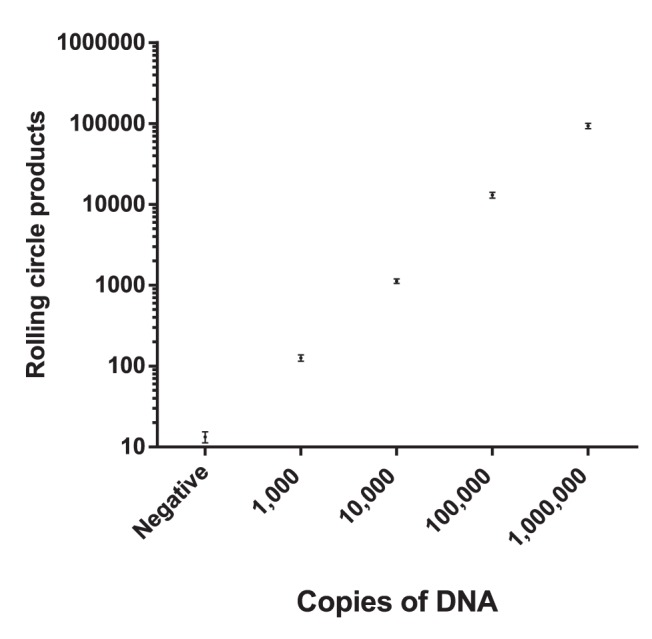
Analytical sensitivity of the method. Short synthetic 5′ biotinylated DNA templates are serially diluted to assess the analytical sensitivity of our assay. The y-axis shows the number of rolling circle products (RCPs) and the x-axis the copy number of a synthetic biotinylated DNA target. The negative sample is a no template control. Error bars ±1 s.d.; n = 3.

Before testing the assay on patient samples the analytical sensitivity was estimated by preparing a 1∶10 dilution series from 10^6^ to 10^3^ copies of a short synthetic biotinylated target (5′ Biotin -TTCATAGTAATTATCATTTGGTTAAATTG-3′). This target is a not an ideal substitute for the real viral genome, but should be a relatively good substitute for the cDNA copy of the genome. Thus, any loss in cDNA synthesis efficiency is not taken into account with this target. C2CA was performed as described in materials and methods and 2.5 µl were analyzed in the microfluidic detection system. With the current setup the assay has a limit of detection of 1,000 molecules and a dynamic range of at least four magnitudes (Figure 3). The amount of target was shown to be linearly correlated to the number of RCPs. These properties allow for a quantitative assay with sufficient sensitivity for analysis of clinical samples, since clinical samples from rotavirus patients usually contain viral loads exceeding this limit of detection.

### Clinical sensitivity

**Figure 4:**
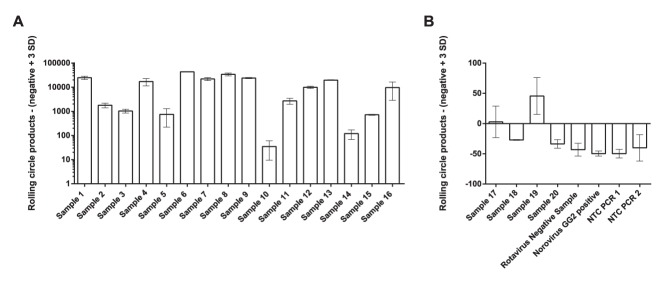
Analysis of patient samples. cDNA was prepared from 22 patient samples collected at Uppsala University hospital. (A) PCR positive samples. (B) PCR negative samples. Plotted is the number of rolling circle product - (negative+3 SD) on the y-axis and patient samples on the x-axis. Error bars ±1 s.d.; n = 2.

One of the most important parameters for a diagnostic method is, next to specificity, clinical sensitivity. Ideally this should be 100%, but most molecular methods that are based on nucleic acid detection have a high risk of generating false negative results for highly variable viruses, such as rotavirus. Mismatches in probes or primers can result in a decreased or absent output signal. It is often difficult to combine several probes or primers to achieve complete coverage of possible strain variations. To investigate the tolerance for sequence variations using our padlock probe based approach, we tested samples from 20 patients diagnosed as rotavirus infected, and two patient samples diagnosed as rotavirus negative. The rotavirus positive samples contained samples from genogroup I (sample 2, 3, 4, 5, 17, 18 and 19) and genogroup II (sample 8, 9, and 10) of VP6. The other rotavirus positive samples could not be classified due to too short sequence information available. cDNA synthesis was confirmed by PCR followed by gel electrophoresis. All rotavirus samples positive by PCR were successfully identified (Figure 4A). All rotavirus negative samples and no target control samples were scored negative by the reported assay and did not show any visible band on the gel in the PCR (Figure 4B). Samples number 17, 18, 19 and 20, all previously identified as rotavirus positive by either ELISA (IDEIA Rotavirus assay, Dako, Denmark) (sample 20) or VOCMA PCR (sample 17–19) [12], did not show any visible bands by PCR, while sample 19 yielded a clearly positive signal by our assay (Figure 4B). In this study, a variation tolerant and specific assay with good sensitivity for detecting rotavirus of genogroup I and II VP6 was demonstrated.

### Dilution series of clinical samples

To assess the robustness of the test in terms of analytical sensitivity and to determine the limit of quantification in clinical samples we performed a dilution series on sample number 1, 4, 6, 8, 10 and 14. Sample 1, 4 and 6 were even detectable when diluting 1∶1,000 whereas sample 8, 10 and 14 could only be diluted 100 times to be clearly above detection limit (Figure S3). A linear quantitative response could be seen for the 1∶10 and 1∶100 dilutions, while the 1∶1,000 dilution falls outside the quantitative range. The higher values of RCPs, compared to the undiluted samples, are due to the larger volume measured (40 µl instead of 2.5 µl).

## Discussion

We hereby present a new rapid and sensitive assay for the detection of rotavirus in clinical samples with a total assay time of about 3.5 hours.

PCR assays are commonly used for nucleic acid detection, but they are limited in the degree of multiplexity and require two spatially separated recognition events, one for each primer. These properties render PCR assays less suitable for the detection of highly variable viruses. In contrast, padlock probes can be easily multiplexed by increasing the number of padlock probes and require only one recognition site. Even the emergence of new strain variants can be easily incorporated without tedious optimizations into the assay by simply designing a matching padlock probe and adding it to the existing pool of padlock probes.

Four out of 20 rotavirus positive samples did not yield any visible PCR product on the agarose gel. This could be due to failed cDNA synthesis, too low quality of the dsRNA or due to nonmatching primers. The specific primer for cDNA synthesis used in this assay could be replaced by random hexamers to make cDNA synthesis sequence independent, and thus create an assay even more tolerant to sequence variation. Sample 19 was detected by our padlock-based method, but not by PCR. This might be due to the fact that PCR requires two primers instead of one and is thereby less variation tolerant. The padlock-based assay is very versatile in forms of read-out. The microfluidic setup combined with a confocal microscope allows for a digital, quantitative read-out, but in many cases a yes or no answer is sufficient. Thus simpler read-outs, that are less quantitative, can be applied. One example of such a readout is the combination of padlock probes with the detection of horseradish peroxidase in a photometer [7]. Other possibilities are using magnetic nanobeads to bind rolling circle products and measure the change in Brownian rotation frequency [14] or using molecular beacons for real-time detection [15].

To summarize, we demonstrated for the first time the detection of a highly variable dsRNA virus using padlock probes.

## Supporting Information

Figure S1 **Alignment of rotavirus sample sequences.** The aligned region contains the target site for the designed padlock probes (nucleotide position 35–63). The alignment was created using Geneious version 6.1 created by Biomatters. Disagreements to the consensus sequences are highlighted in color.
(TIF)

Figure S2 **Evaluation of padlock probes.** To investigate the effect of an increasing number of padlock probes on rolling circle product (RCP) formation different numbers of probes were added to the same concentration of a short synthetic 5′ biotinylated DNA template, ligated and amplified by C2CA. The negative control is a no template control. Forty µl of sample were analyzed. Plotted values are the number of measured RCPs. Error bars ±1 s.d.; n = 2.
(EPS)

Figure S3 **Dilution series of patient samples.** Six patient samples were diluted from 1∶10 to 1∶1,000 in order to show a linear correlation between target concentration and the number of detected rolling circle products (RCPs). Plotted is the number of rolling circle product - (negative+3 SD) on the y-axis and the dilution factor on the x-axis. Error bars ±1 s.d.; n = 2.
(EPS)
