# Supplementary figures and images for: Detection of Rotavirus Using Padlock Probes and Rolling Circle Amplification
Source: PLoS One. 2014 Nov 4;9(11):e111874. doi: 10.1371/journal.pone.0111874 (PMC4219791; doi:10.1371/journal.pone.0111874)

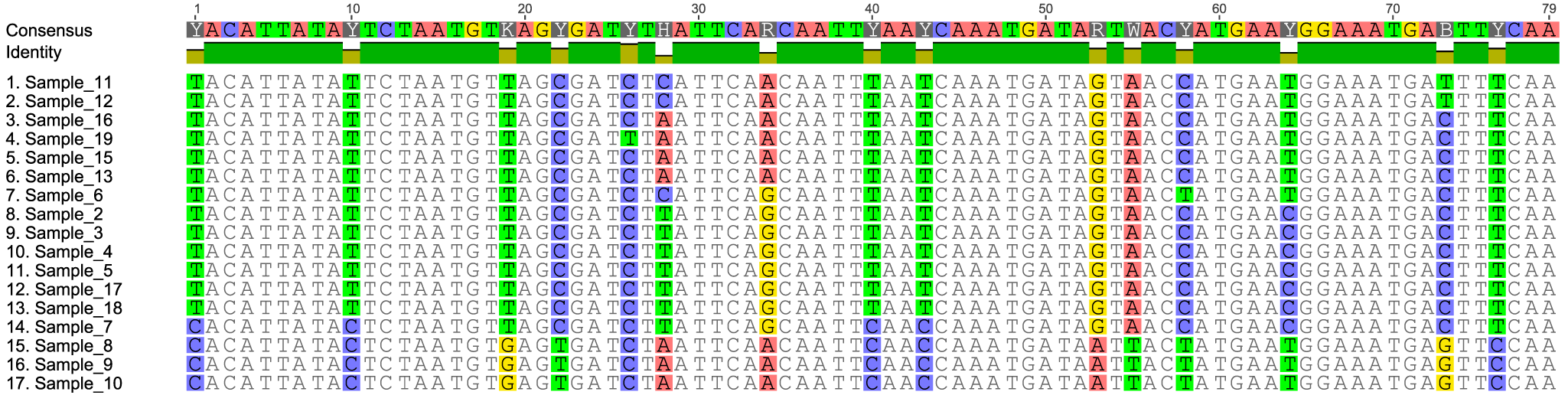

Supplement: Figure S1 — Alignment of rotavirus sample sequences. The aligned region contains the target site for the designed padlock probes (nucleotide position 35–63). The alignment was created using Geneious version 6.1 created by Biomatters. Disagreements to the consensus sequences are highlighted in color. (TIF) [file pone.0111874.s001.tif]
